# Paired Whole-Genome Sequencing of Scalp Angiosarcoma and Matched Lung Metastasis Reveals Common Clonal Origin and Lung-Specific Evolution

**DOI:** 10.3390/diagnostics16172824

**Published:** 2026-09-02

**Authors:** Green Hong, Yooyoung Chong, Joo-Eun Lee, Hyun-Yi Kim, Dahye Lee, Young Lee, Min-Kyung Yeo, Chaeuk Chung

**Affiliations:** 1 Department of Pulmonary and Critical Care Medicine, Chungnam National University Hospital, 282 Munhwa-ro, Jung-gu, Daejeon 35015, Republic of Korea; ihongreen@naver.com; 2 Department of Internal Medicine, College of Medicine, Chungnam National University, 266 Munhwa-ro, Jung-gu, Daejeon 35015, Republic of Korea; ziczi02@naver.com; 3 Department of Thoracic & Cardiovascular Surgery, College of Medicine, Chungnam National University, 266 Munhwa-ro, Jung-gu, Daejeon 35015, Republic of Korea; yooychong@gmail.com; 4 Department of Biomedical Research Institute, Chungnam National University Hospital, 282 Munhwa-ro, Jung-gu, Daejeon 35015, Republic of Korea; jooeunlee83@gmail.com; 5 NGeneS Inc., Sangnok-gu, Asan 15495, Republic of Korea; hykim@ngenes.co.kr; 6 Department of Dermatology, College of Medicine, Chungnam National University, 266 Munhwa-ro, Jung-gu, Daejeon 35015, Republic of Korea; resina20@cnu.ac.kr; 7 Department of Pathology, College of Medicine, Chungnam National University, 266 Munhwa-ro, Jung-gu, Daejeon 35015, Republic of Korea

**Keywords:** angiosarcoma, pulmonary metastasis, cystic lung disease, whole genome sequencing

## Abstract

**Background and Clinical Significance:** Cutaneous angiosarcoma frequently metastasizes to the lungs, where it may rarely present as diffuse cystic lung disease with recurrent pneumothorax, resulting in substantial diagnostic difficulty. We report a case of pulmonary metastatic cutaneous angiosarcoma in which paired whole-genome sequencing (WGS) of the primary and metastatic lesions was performed to clarify clonal origin and characterize metastatic evolution. **Case Presentation:** A 65-year-old man with recurrent right-sided pneumothorax and progressive bilateral cystic lung lesions underwent skin and lung biopsies. Histopathological examination and immunohistochemistry established the diagnosis of cutaneous angiosarcoma with pulmonary metastases. Paired WGS was performed on matched scalp and lung tumor specimens to evaluate shared and lesion-specific genomic alterations, pathway enrichment, and copy-number changes. Histopathology confirmed metastatic angiosarcoma involving the lungs. WGS identified 128 shared somatic alterations, supporting a common clonal origin, together with lung-specific and skin-specific mutations indicative of continued genomic divergence. Recurrent alterations involving POT1 and FLT4 were preserved in both lesions, whereas additional POT1 and TP53 alterations were detected only in the pulmonary metastasis. Pathway analysis demonstrated preferential enrichment of IGF1–mTOR, RAS, and WNT/LRP6 signaling in the metastatic lesion, while Gene Ontology analysis suggested functional divergence associated with metastatic progression. **Conclusions:** Pulmonary metastatic angiosarcoma should be considered in patients presenting with unexplained diffuse cystic lung disease and recurrent pneumothorax, particularly when pathological findings are inconclusive. Paired WGS complemented conventional histopathology by confirming the metastatic origin and providing insights into clonal evolution and lesion-specific molecular alterations, highlighting its potential value in the investigation of rare metastatic malignancies.

## 1. Introduction

Cystic lung diseases encompass a heterogeneous spectrum of disorders, ranging from primary entities such as lymphangioleiomyomatosis (LAM), pulmonary Langerhans cell histiocytosis, Birt–Hogg–Dubé syndrome, and lymphocytic interstitial pneumonia to secondary causes, including infections and, less commonly, metastatic malignancies [1]. In clinical practice, multiple thin-walled pulmonary cysts are usually investigated within the framework of primary cystic lung diseases; however, metastatic neoplasms may occasionally present with a similar radiologic appearance and pose a diagnostic challenge [2].

Angiosarcoma is a rare and highly aggressive malignant neoplasm of vascular endothelial origin, accounting for less than 1% of soft-tissue sarcomas. Approximately 20–45% of patients present with metastatic disease at diagnosis, most commonly involving the lungs [3,4,5]. Pulmonary metastases of angiosarcoma exhibit a notably broad imaging spectrum that distinguishes them from most other metastatic malignancies. While solid bilateral nodules may be observed, a characteristic and often dominant pattern is the presence of multiple thin-walled cystic or bullous lesions, sometimes accompanied by ground-glass opacities or interlobular septal thickening from lymphangitic spread [6]. The cysts are thought to result from infiltrative growth of tumor cells along alveolar walls, with eventual remodeling of the airspace into dilated cavities [7]. These lesions may be further complicated by spontaneous pneumothorax, hemothorax, or hemoptysis, which can precede recognition of the underlying malignancy and delay diagnosis [7,8].

Recent genomic studies have revealed substantial molecular heterogeneity in angiosarcoma, identifying distinct mutational processes, including ultraviolet radiation-associated mutational signatures, variable tumor mutation burdens, and potentially actionable genomic alterations. However, most available data are derived from analyses of primary tumors, and the genomic evolution underlying metastatic dissemination remains poorly understood [9,10]. To our knowledge, whole-genome sequencing (WGS) directly comparing paired primary and metastatic angiosarcoma lesions from the same patient has rarely been reported. Such paired analyses enable the reconstruction of clonal relationships and identification of metastasis-associated genomic alterations that cannot be inferred from primary tumors alone. WGS provides a comprehensive approach to characterize somatic mutations, structural variants, copy-number alterations, and mutational signatures across the entire genome, offering a unique opportunity to investigate tumor evolution during metastatic progression.

Here, we report a patient who presented with refractory cystic lung disease and recurrent pneumothorax and was ultimately diagnosed with pulmonary metastases from cutaneous angiosarcoma of the scalp. We performed paired WGS of the primary scalp tumor and metastatic pulmonary lesion to define their clonal relationship and characterize genomic alterations associated with pulmonary metastasis.

## 2. Case Description

**Figure 1:**
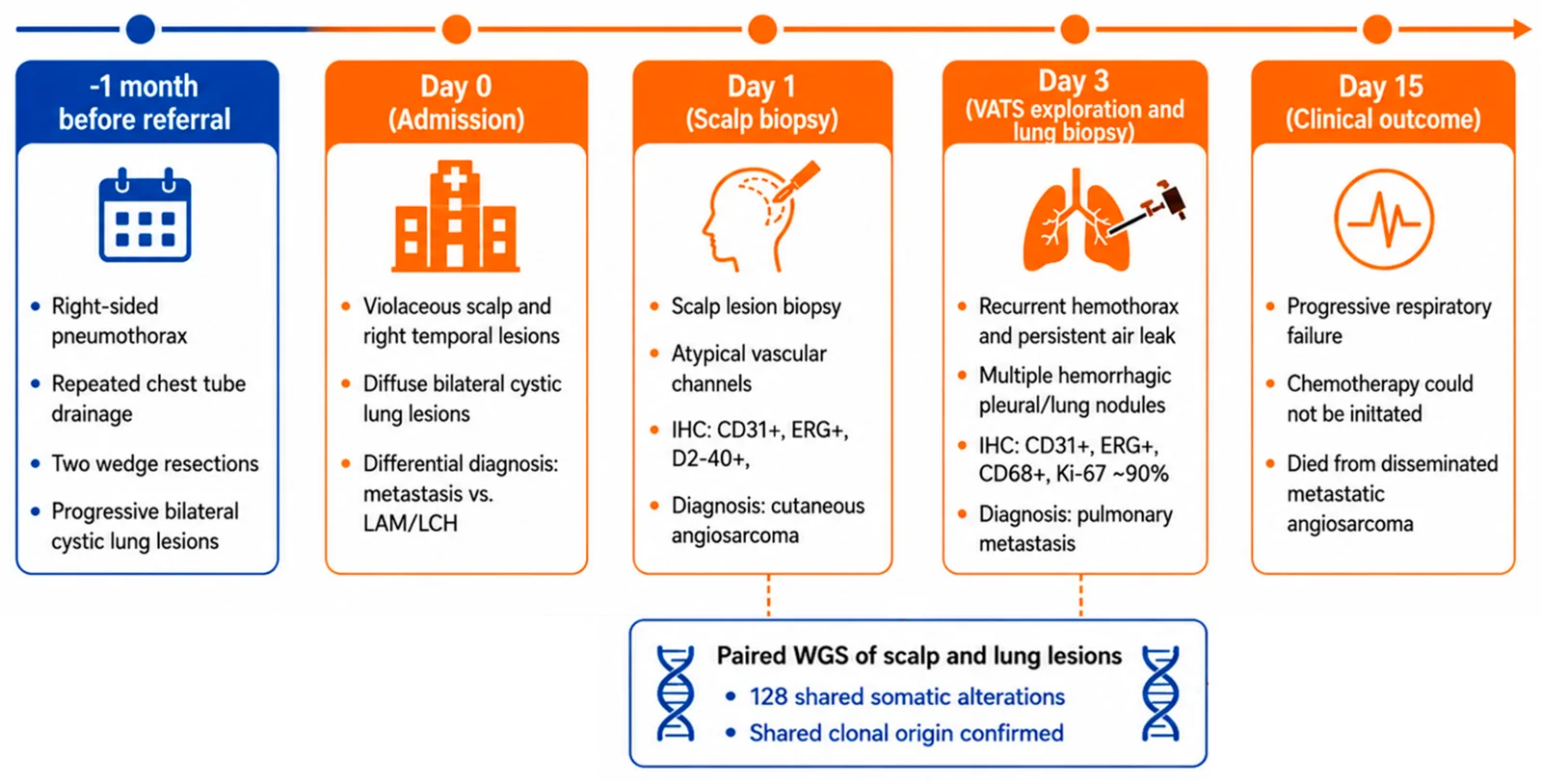
Clinical timeline and diagnostic workflow. Timeline of the patient’s clinical course, including recurrent pneumothorax, diagnostic evaluation, scalp biopsy, video-assisted thoracoscopic surgery (VATS) lung biopsy, paired WGS, and clinical outcome. Paired WGS identified 128 shared somatic alterations between the primary cutaneous and pulmonary metastatic lesions, supporting a common clonal origin.

**Figure 2:**
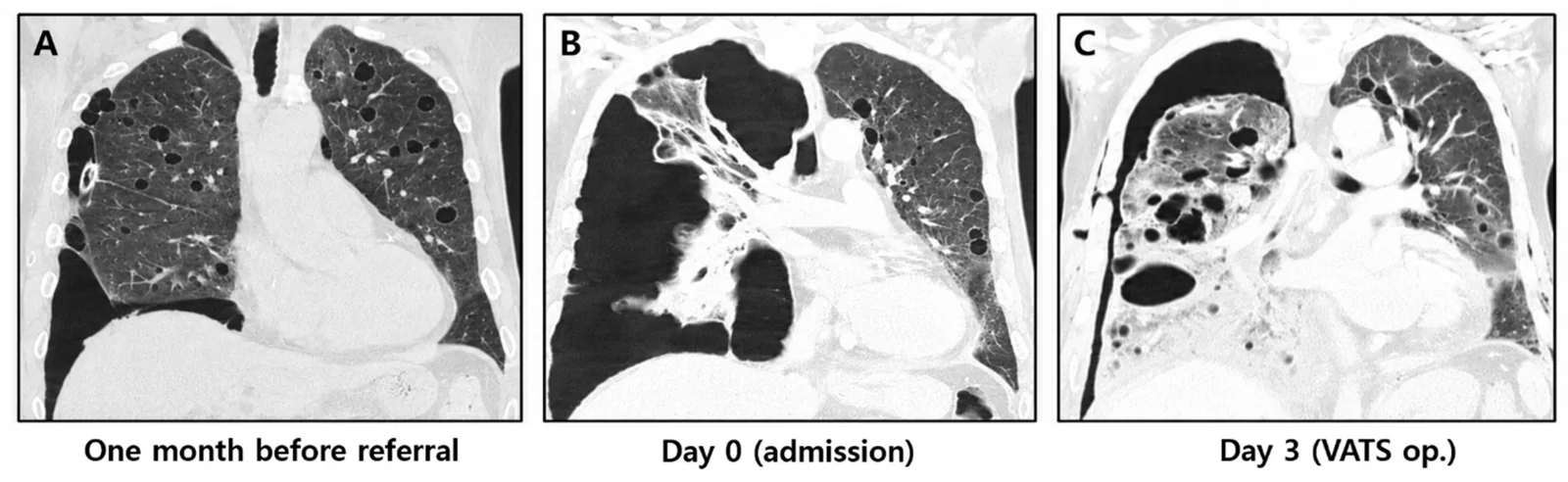
Evolution of pulmonary cystic lesions on chest computed tomography (CT): (**A**) Initial chest CT at the outside hospital demonstrating multiple bilateral thin-walled cystic lung lesions with a right-sided pneumothorax. (**B**) Admission CT showing recurrent right-sided pneumothorax with progression of the diffuse cystic lesions. (**C**) Follow-up CT demonstrating rapid progression of extensive cystic and hemorrhagic pulmonary lesions before thoracoscopic biopsy confirmed pulmonary metastatic angiosarcoma.

A 65-year-old male was referred to our institution for evaluation of a persistent right-sided pneumothorax that had been unresponsive to prior interventions. The overall clinical course and diagnostic workup are summarized in Figure 1. One month earlier, at an outside hospital, he had undergone repeated chest tube drainage followed by two wedge resections of the right lung, but the pneumothorax recurred shortly thereafter, and follow-up imaging demonstrated progressive bilateral cystic lung lesions (Figure 2A). Histopathological examination of the previously resected lung tissue had been interpreted as showing no definitive evidence of Langerhans cell histiocytosis, LAM, or infection. Desmin immunostaining was negative, but additional immunohistochemical workup for vascular neoplasms had not been performed at that time, as angiosarcoma was not initially considered in the differential diagnosis. No specific diagnosis was reached prior to transfer. 

**Figure 3:**
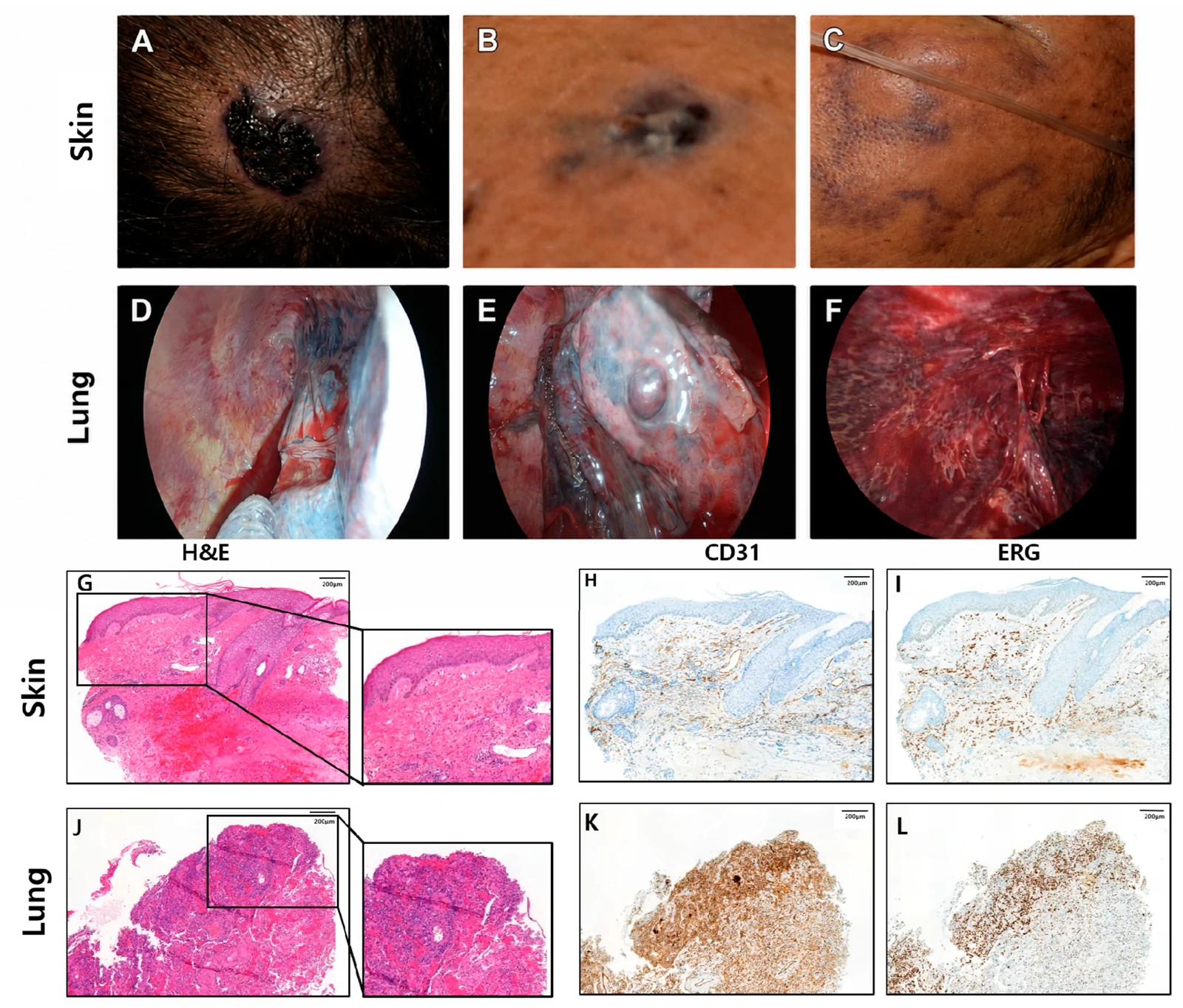
Clinical, thoracoscopic, and histopathological findings of cutaneous angiosarcoma with pulmonary metastasis: (**A**) Long-standing violaceous nodular lesion on the scalp. (**B**) Violaceous lesion on the temple. (**C**) Purpuric/violaceous lesion involving the cheek. (**D**–**F**) VATS demonstrating multiple hemorrhagic nodules involving the pleura and lung surface. (**G**) Hematoxylin and eosin (H&E) staining of the scalp biopsy showing irregular anastomosing vascular channels lined by atypical endothelial cells. (**H**,**I**) Immunohistochemistry of the scalp lesion demonstrating positivity for CD31 and ERG. (**J**) H&E staining of the lung biopsy demonstrating infiltrative vascular proliferation morphologically similar to the primary cutaneous lesion. (**K**,**L**) Immunohistochemistry of the lung lesion demonstrating positivity for CD31 and ERG, confirming pulmonary metastatic angiosarcoma.

On admission, physical examination revealed violaceous nodules and patches involving the scalp and temple (Figure 3A,B). The patient reported a long-standing scalp papule; although he was aware of its presence, he was unable to recall its exact onset, and the lesion had gradually enlarged over time without prior medical evaluation. In the weeks preceding transfer, a new violaceous patch resembling petechiae had developed on the temple and cheek (Figure 3C). Given these atypical cutaneous findings in the context of unexplained cystic lung disease, skin biopsies were performed to evaluate for an underlying malignancy.

Chest computed tomography (CT) performed on admission demonstrated multiple thin-walled, air-filled cystic lesions diffusely distributed throughout both lungs, accompanied by a recurrent right-sided pneumothorax (Figure 2B). Given the predominance of diffuse cystic lesions, the radiologic appearance closely mimicked a primary cystic lung disease such as LAM, although pulmonary metastatic disease remained in the differential diagnosis. During hospitalization, the patient developed recurrent hemothorax and persistent air leakage, eventually resulting in respiratory failure and hemodynamic instability. Thoracoscopic exploration revealed multiple hemorrhagic nodules involving the pleura and lung parenchyma, prompting biopsy of the pulmonary lesions (Figure 2C and Figure 3D–F).

Histopathological examination of the scalp and temple biopsies demonstrated dermal proliferation of irregular, anastomosing vascular channels lined by atypical endothelial cells, consistent with cutaneous angiosarcoma. Immunohistochemical staining showed positivity for CD31, ERG (ETS-related gene), and D2-40, with focal CD34 positivity and CD30 negativity (Figure 3G–I). Histopathological evaluation of the lung biopsy revealed infiltrative vascular proliferation morphologically analogous to that observed in the cutaneous lesions. Tumor cells were positive for CD31, ERG, and CD68, with a markedly elevated Ki-67 labeling index of approximately 90% (Figure 3J–L). Taken together, these findings established the diagnosis of pulmonary metastases from cutaneous angiosarcoma of the scalp.

Given the extensive pulmonary involvement and advanced metastatic disease, curative surgical resection was not feasible. Although systemic chemotherapy was planned, the patient’s clinical condition deteriorated rapidly because of recurrent intrathoracic hemorrhage and progressive respiratory failure. He ultimately died from complications of disseminated angiosarcoma before anticancer treatment could be initiated.

## 3. Methods

Genomic DNA was extracted from formalin-fixed, paraffin-embedded (FFPE) tissues using an automated system following the manufacturer’s protocol, which included proteinase K digestion and high-temperature incubation to ensure effective tissue lysis. DNA quantity and quality were assessed with spectrophotometric and electrophoretic methods before preparing the sequencing library. Sequencing libraries were prepared using a standard protocol and subjected to paired-end sequencing on a high-throughput Illumina platform.

Sequencing reads were aligned to the human reference genome GRCh38 using the Burrows–Wheeler alignment algorithm. Duplicate reads were marked, and base quality score recalibration was performed according to the Genome Analysis Toolkit (GATK) Best Practices workflow. The same matched non-tumor lung sample was used as the germline control for both the primary skin lesion and the metastatic lung lesion.

Somatic single nucleotide variants (SNVs) and small insertions and deletions (indels) were identified using GATK Mutect2 (v4.6.2.0) in paired tumor-normal mode. The gnomAD population allele frequency resource and the 1000 Genomes-derived panel of normals were incorporated during variant calling to facilitate exclusion of common germline variants and recurrent technical artifacts. Raw calls were subsequently processed using GATK FilterMutectCalls. For downstream analysis, variants were retained when the tumor sequencing depth was ≥20 reads, the tumor variant allele frequency (VAF) was ≥0.05, the corresponding VAF in the matched normal sample was <0.02, and the variant was not flagged by the panel of normals. VAF was defined as the fraction of sequencing reads supporting the alternate allele among reads covering the corresponding genomic position. Functional annotation of filtered variants was performed using GATK Funcotator with the hg38 somatic annotation data-source bundle v1.8.hg38.20230908s.

Genome-wide sequencing coverage and alignment quality were evaluated using the recalibrated binary alignment map (BAM) files. Mean genome-wide sequencing depths were 20.36× for the metastatic lung lesion, 22.82× for the primary skin lesion, and 25.18× for the matched non-tumor lung tissue sample, with corresponding median depths of 20×, 23×, and 25×, respectively. The proportions of the genome covered at ≥20× were 56.28%, 68.18%, and 75.11%, respectively. Primary mapping rates were 99.86%, 99.79%, and 99.81%, respectively, and duplicate read fractions determined using Picard MarkDuplicates were 8.94%, 12.96%, and 12.24%, respectively.

Tumor cellularity and ploidy were independently evaluated using Sequenza v3.0.0, which jointly models tumor-to-normal sequencing-depth ratios and B-allele frequencies at informative germline loci. Analyses were restricted to autosomes, and cellularity and ploidy were evaluated over ranges of 0.01–1.00 and 1.0–7.0, respectively. For the primary skin lesion, the maximum-posterior cellularity estimate was 0.05; however, the confidence region extended from 0.01 to 1.00, indicating that tumor cellularity could not be robustly determined. The corresponding ploidy estimate was 1.7 (95% confidence region, 1.0–2.6). For the metastatic lung lesion, the maximum-posterior cellularity estimate was 0.06 (95% confidence region, 0.02–0.10), whereas the corresponding ploidy estimate of 1.0 was poorly constrained (95% confidence region, 1–3.1).

Genome-wide copy-number alterations were analyzed using CNVkit v0.9.10 in whole-genome sequencing mode. For each tumor sample, read-depth profiles were normalized against the matched non-tumor tissue sample using CNVkit reference-based normalization and systematic coverage-bias correction. Normalized bin-level log2 tumor-to-normal copy ratios were segmented to identify regions of relative copy-number gain and loss. Because the Sequenza-derived cellularity estimates, particularly for the primary skin lesion, were associated with substantial uncertainty, CNVkit profiles were interpreted as relative copy-number alterations and were not converted to purity-corrected absolute copy numbers.

## 4. Results

Whole-genome sequencing identified 128 shared somatic alterations between the primary scalp tumor and pulmonary metastasis, whereas 361 and 617 alterations were unique to the pulmonary metastasis and primary scalp tumor, respectively (Supplementary Figure S1). The overall mutational spectrum was comparable between the two lesions, with missense mutations representing the predominant variant class in both tumors (Supplementary Figure S2). Several identical coding alterations were shared between the primary and metastatic lesions, including a protection of telomeres 1 (POT1) frameshift deletion, c.428_440delATATGTCACCGTC (p.H143fs); a Fms-related receptor tyrosine kinase 4 (FLT4) missense variant, c.3181G>T (p.D1061Y); a natriuretic peptide receptor 1 (NPR1) variant, c.2812_2813GG>AA (p.G938K); and a sortilin-related VPS10 domain-containing receptor 3 (SORCS3) variant, c.3140_3141CC>TT (p.A1047V).

Cleavage and polyadenylation specificity factor 1 (CPSF1) was also altered in both lesions, although at different residues (p.R1049G in the primary tumor and p.T697M in the pulmonary metastasis). The pulmonary metastasis retained the POT1 p.H143fs alteration present in the primary tumor and additionally harbored a POT1 p.T187I missense variant within the N-terminal OB2 telomeric DNA-binding domain. The shared FLT4 p.D1061Y alteration lies within the VEGFR3 (vascular endothelial growth factor receptor 3) tyrosine kinase domain; however, this specific substitution has not been established as an activating or oncogenic mutation, and its functional significance therefore remains uncertain. In contrast, a TP53 missense mutation (c.655C>T; p.P219S) was detected only in the pulmonary metastasis (Supplementary Figure S3).

Pathway analysis demonstrated distinct pathway alteration profiles between the paired lesions. The pulmonary metastasis showed alterations affecting the insulin-like growth factor 1–mechanistic target of rapamycin (IGF1–mTOR), rat sarcoma (RAS), tumor protein p53 (TP53), and WNT/low-density lipoprotein receptor-related protein 6 (WNT/LRP6) pathways, whereas the primary tumor showed enrichment of transforming growth factor beta (TGF-β), TP53/hypoxia, and WNT signaling pathways (Supplementary Figure S4). Gene Ontology (GO) biological process annotation profiles showed that genes with alterations shared between the two lesions were frequently associated with cell growth, wound healing, and cytoskeleton organization. Lung-specific altered genes were commonly annotated to membrane potential regulation, peptide hormone responses, cilium organization, and protein phosphorylation, whereas skin-specific altered genes were frequently annotated to cell development, axonogenesis, and cell cycle-related processes (Supplementary Figure S5). Genome-wide copy-number analysis demonstrated broadly similar chromosomal alteration patterns between the paired lesions, although several lesion-specific copy-number gains and losses were also identified (Supplementary Figure S6). In addition, Circos plots were generated to provide an integrated visualization of genome-wide genomic alterations, including somatic variants and copy-number changes, in the primary scalp tumor and pulmonary metastasis (Supplementary Figure S7). Detailed lists of somatic variants, lung- and skin-specific copy-number variants, and oncogenic pathway-related variants are provided in Supplementary Tables S1–S4, respectively.

## 5. Discussion

This case illustrates the diagnostic challenge of cystic pulmonary metastases from cutaneous angiosarcoma and demonstrates how paired WGS can complement conventional pathology by clarifying metastatic origin and providing insight into tumor evolution.

From a pulmonary perspective, this case illustrates a recurring diagnostic challenge. The initial presentation—refractory pneumothorax accompanied by bilateral thin-walled cystic lung lesions—prompted a diagnostic workup focused on primary cystic lung diseases such as LAM and pulmonary Langerhans cell histiocytosis. Two wedge resections performed at an outside institution failed to establish a diagnosis, and vascular endothelial markers were not included in the initial immunohistochemical evaluation because angiosarcoma was not suspected. Similar diagnostic delays have been reported in patients with pulmonary metastatic angiosarcoma, in whom recurrent pneumothorax often precedes recognition of the underlying malignancy [7]. In retrospect, the patient harbored a long-standing scalp lesion that remained unevaluated despite gradual enlargement, illustrating how the cutaneous primary tumor may be overlooked when respiratory manifestations dominate the clinical presentation. Accordingly, metastatic angiosarcoma should be considered in elderly patients with unexplained cystic lung disease, particularly in the setting of recurrent pneumothorax, hemothorax, or nondiagnostic pathology. Careful skin examination and inclusion of vascular markers such as CD31 and ERG may facilitate earlier diagnosis.

Recent genomic studies have demonstrated that angiosarcoma exhibits substantial molecular heterogeneity while consistently identifying recurrent alterations involving TP53, MYC, KDR, POT1, ATRX, PIK3CA, FLT4, PTPRB, and components of the RAS pathway [11]. However, because most studies have analyzed unrelated patient cohorts rather than matched primary and metastatic specimens, the temporal sequence of these genomic alterations during metastatic progression remains poorly understood.

In our patient, paired WGS independently confirmed the common clonal origin of the pulmonary lesions while simultaneously demonstrating continued molecular divergence after metastatic dissemination. The persistence of recurrent angiosarcoma-associated alterations, particularly POT1 and FLT4, suggests that disruption of telomere maintenance and angiogenic signaling represents an early event established in the founding clone and preserved throughout metastatic progression. In contrast, the emergence of additional alterations in the pulmonary metastasis, including a second POT1 mutation and TP53 alteration, supports continued clonal diversification rather than simple expansion of an unchanged metastatic clone (Supplementary Figure S3). Although the biological significance of these individual alterations remains uncertain, the overall genomic architecture supports progressive tumor evolution following dissemination.

**Figure 4:**
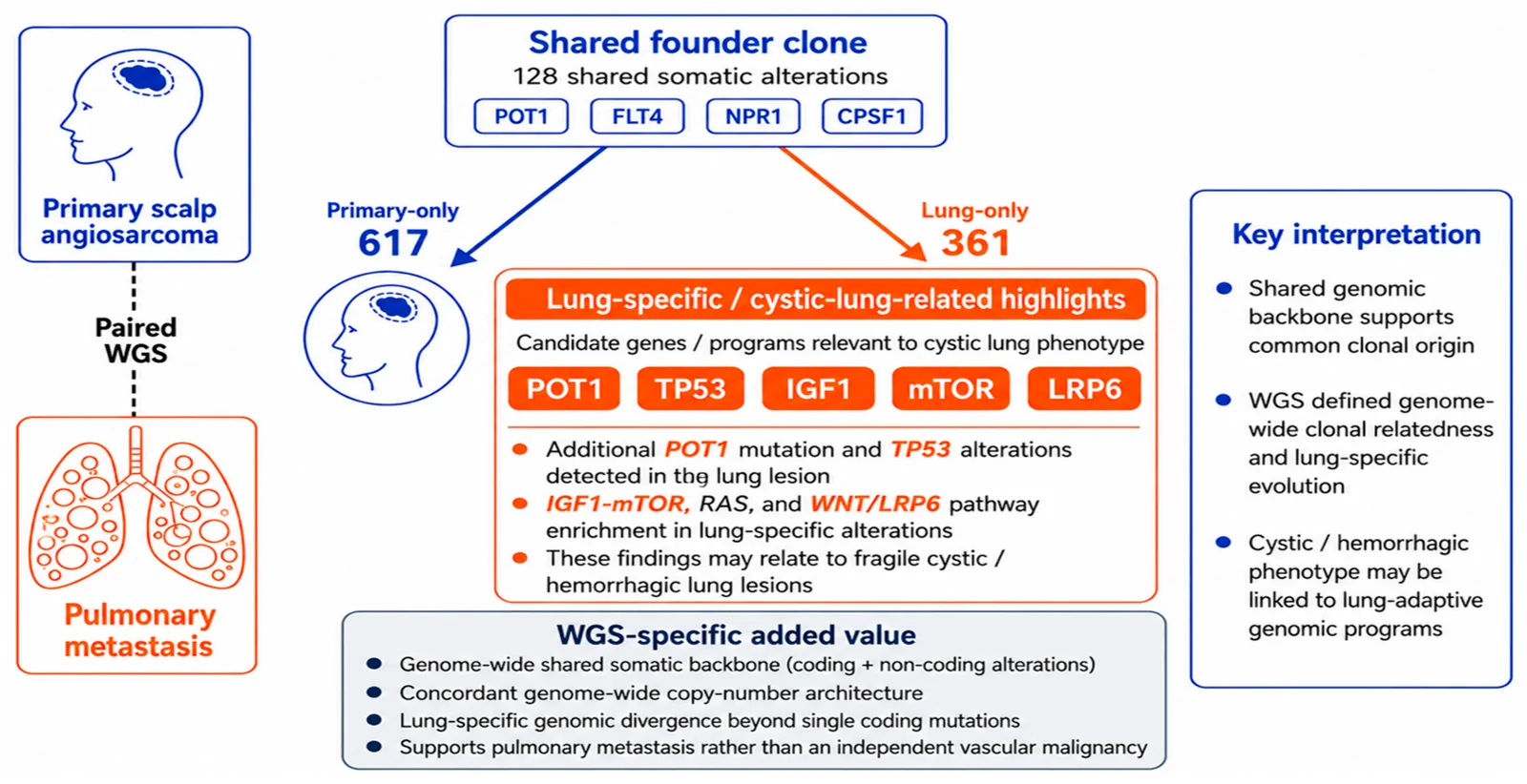
Schematic summary of paired WGS findings in primary cutaneous angiosarcoma and pulmonary metastasis. Paired WGS demonstrated a shared founder clone with 128 common somatic alterations, supporting a common clonal origin of the primary scalp tumor and pulmonary metastasis. The metastatic lung lesion harbored lung-specific alterations, including an additional POT1 mutation and a TP53 alteration, together with preferential involvement of the IGF1–mTOR, RAS, and WNT/LRP6 signaling pathways. Overall, these findings suggest preservation of a common genomic backbone followed by branched molecular divergence of the primary and metastatic lesions.

Functional analyses further reinforced this concept. Rather than simply accumulating additional mutations, the pulmonary metastasis demonstrated preferential involvement of signaling pathways related to IGF1–mTOR, RAS, and WNT/LRP6, whereas the primary cutaneous lesion showed greater enrichment of TGF-β, hypoxia, and WNT-associated pathways. Gene Ontology annotation profiles similarly suggested functional divergence, with shared alterations frequently associated with cell growth and wound healing; lung-specific alterations commonly annotated to processes involving membrane potential regulation, peptide hormone responses, cilium organization, protein phosphorylation, and cell adhesion; and skin-specific alterations frequently associated with cell development and cell cycle regulation. Although these analyses are exploratory and derived from a single patient, the presence of substantial numbers of private alterations in both lesions, together with their distinct functional profiles, is more consistent with branched evolution from a shared ancestral clone followed by continued and independent genomic evolution at each anatomical site (Figure 4). Thus, the relative numbers of lesion-specific alterations should not be interpreted as evidence of sequential mutation accumulation predominantly in the pulmonary metastasis or as an indicator of the timing of metastatic dissemination. Comparison of UV-associated mutational signatures between the primary scalp tumor and pulmonary metastasis could provide additional insight into their evolutionary history; however, mutational signature analysis was not performed in the present study, precluding conclusions regarding UV-related mutagenesis or its potential relationship to the timing of metastatic dissemination.

Although no targeted therapy could be administered because of the patient’s rapid clinical deterioration, several genomic findings may have potential therapeutic relevance. Given the vascular origin of angiosarcoma, anti-angiogenic strategies targeting VEGF/VEGFR signaling have been investigated in advanced disease. Pazopanib represents a systemic treatment option, while other VEGF/VEGFR-targeting agents, including regorafenib, sorafenib, sunitinib, and bevacizumab, may be considered in selected clinical settings. In our case, the shared FLT4 p.D1061Y variant is of particular interest because FLT4 encodes VEGFR3, providing a potential biological link to angiogenic signaling. However, the functional significance of this specific variant remains uncertain, and it should not be interpreted as a predictive biomarker for VEGFR-targeted therapy. In contrast to anti-angiogenic approaches, evidence supporting PI3K/AKT/mTOR-directed therapies in angiosarcoma remains limited and investigational. The alterations contributing to mTOR pathway enrichment in the pulmonary metastasis may provide biological insight but do not establish therapeutic sensitivity. Thus, these genomic findings should be considered hypothesis-generating rather than predictive of treatment response [12].

Several limitations should be acknowledged. Genomic analyses were performed using FFPE-derived tissue, and matched peripheral blood was unavailable for definitive germline filtering. In addition, conclusions regarding metastatic evolution are inherently limited by the analysis of a single patient. Nevertheless, whereas previous genomic studies have primarily characterized interpatient molecular heterogeneity in angiosarcoma, our paired whole-genome analysis provides a rare intrapatient perspective by demonstrating how a conserved founder clone can give rise to biologically divergent metastatic disease. These findings further illustrate the complementary role of comprehensive genomic profiling alongside conventional histopathology in confirming metastatic origin and reconstructing tumor evolution.

In conclusion, cystic pulmonary metastases from cutaneous angiosarcoma can closely mimic primary cystic lung disease and result in substantial diagnostic delay. In elderly patients presenting with unexplained cystic lung lesions, particularly when accompanied by recurrent or hemorrhagic pneumothorax, metastatic angiosarcoma should be included in the differential diagnosis, and careful skin examination should be performed. Our case further demonstrates that paired whole-genome sequencing can extend beyond diagnostic confirmation by revealing clonal relationships and metastatic evolution, providing biological insights that may ultimately facilitate precision approaches for this rare and aggressive malignancy.

## Data Availability

All data generated or analyzed during this study are included in this published article and its Supplementary Information Files. Raw data files are available upon request from the corresponding author.

## Supplementary Materials

The following supporting information can be downloaded at https://www.mdpi.com/article/10.3390/diagnostics16172824/s1, Figure S1: UpSet plot of shared and lesion-specific somatic alterations identified by paired whole-genome sequencing; Figure S2: Variant classification plot of the paired whole-genome sequencing data; Figure S3: Oncoplot of recurrent somatic alterations identified by paired whole-genome sequencing; Figure S4: Treemap of altered signaling pathways identified by paired whole-genome sequencing; Figure S5: Gene Ontology biological process annotation profile of shared and lesion-specific somatic alterations; Figure S6: Genome-wide copy-number alteration profile of the paired lesions; Figure S7: Circos plots of genomic alterations in the scalp and lung lesions; Table S1: Somatic variants identified in the scalp and lung lesions; Table S2: Copy number variants identified in lung lesion; Table S3: Copy-number variants identified in the scalp lesion; Table S4: Oncogenic pathway-related variants identified in the scalp and lung lesions.
